# Meaning of ACR-TIRADS recommendation in favor of follow-up rather than FNAC in thyroid nodules

**DOI:** 10.1007/s13304-024-01886-4

**Published:** 2024-05-21

**Authors:** Andrea Leoncini, Marco Curti, Lorenzo Ruinelli, Pierpaolo Trimboli

**Affiliations:** 1 Servizio Di Radiologia E Radiologia Interventistica, Istituto Di Imaging Della Svizzera Italiana (IIMSI), Ente Ospedaliero Cantonale (EOC), 6900 Lugano, Switzerland; 2 Servizio Di Endocrinologia E Diabetologia, Ospedale Regionale Di Lugano, Ente Ospedaliero Cantonale (EOC), 6900 Lugano, Switzerland; 3 Team Data Science & Research, Area ICT, Ente Ospedaliero Cantonale, 6500 Bellinzona, Switzerland; 4 Clinical Trial Unit, Ente Ospedaliero Cantonale (EOC), Bellinzona, Switzerland; 5 Facoltà Di Scienze Biomediche, Università Della Svizzera Italiana (USI), 6900 Lugano, Switzerland

**Keywords:** Thyroid, Ultrasound, TIRADS, Follow-up

## Abstract

Thyroid Imaging Reporting and Data Systems (TIRADSs) have been largely diffused for their high accuracy in risk stratification of thyroid nodules (TNs) and their selection for fine-needle aspiration cytology (FNAC). The most popular TIRADSs are ACR-, EU-, and K-TIRADS, with some discrepancies each other. One major difference is that ACR-TIRADS includes a recommendation in favor of follow-up in TNs having a major diameter insufficient to indicate FNAC. The present study aimed to explore prevalence and significance of this recommendation. EU- and K-TIRADS were used as comparator. A retrospective series of thyroidectomies was searched according to a pre-defined protocol. The study period was 2019–2023. Preoperative ultrasound images were reviewed by radiologists blinded of clinical data. Matching of TIRADS and histology was performed later. Histology was the gold standard. The study series included 39 TNs classified as category 3, 4, or 5 and assessed for follow-up according to ACR-TIRADS. The overall cancer frequency was 25.6%, being 13% in category 3, 20% in category 4, and 83.3% in category 5. The category assessment according to ACR-, EU-, and K-TIRADS was not significantly different. EU-TIRADS indicated FNAC in 10 TNs of which two cancers and eight benign lesions. K-TIRADS recommended FNAC in 32 TNs of which seven cancers and 25 benign lesions. TNs assessed for follow-up according to ACR-TIRADS are cancer in one-fourth of cases. EU- and, especially, K-TIRADS allow us to select for FNAC cancers, with the burden of non-negligible frequency of unnecessary FNACs.

## Introduction

Thyroid nodule (TN) is a frequent entity often detected incidentally during imaging procedures performed according to non-thyroidal indication [1, 2]. As TNs are generally benign, the initial patient assessment aims to rule out those cases that do not require further diagnostic or therapeutic work-up. In this context, ultrasonography (US) is recognized as the most accurate imaging procedure to assess the risk of malignancy (RoM) of TNs. During the last years, endocrinologists and radiologists have become aware of the high reliability of US-based risk stratification systems proposed by international societies and generally reported as Thyroid Imaging Reporting and Data System (TIRADS). Basically, TIRADSs aim to standardize the assessment of RoM of TNs and the selection of TNs requiring fine-needle aspiration cytology (FNAC). The most popular TIRADSs are ACR-, EU-, and K-TIRADS [3–5]. On the one hand, the literature has demonstrated the accuracy of TIRADSs in stratifying the RoM of TNs without significant differences among systems [6, 7]. On the other hand, some discrepancies among TIRADSs were observed in avoiding “unnecessary” FNAC, such as those biopsies that would not been indicated [8, 9].

Each TIRADS includes categories associated with an estimated RoM. In addition, each TIRADS reports a category-specific TN size threshold above which FNAC is indicated. Remarkably, as illustrated in Table 1, both category RoM and category size threshold (and actions associated) proposed by ACR-, EU-, and K-TIRADS diverge. The major difference that catches the eyes is that ACR-TIRADS includes a recommendation in favor of follow-up in TNs assessed as category 3, 4, and 5 and having a major diameter insufficient to indicate FNAC. This recommendation might be not particularly discrepant with the corresponding category 5 of EU- and K-TIRADS that suggest similar clinical actions (i.e., active surveillance or FNAC in selected cases, respectively). However, recommending follow-up in TNs rated as category 3 and 4 according to ACR-TIRADS has not corresponding suggestion in EU- and K-TIRADS. Since it is active a project endorsed by the most important international societies aimed at creating a universal TIRADS (I-TIRADS) [10], exploring the differences among systems holds a crucial role. In fact, I-TIRADS should be generalizable and applicable in all settings.

**Table 1:** Categories and actions included in the ACR-, EU-, and K-TIRADS

Category	ACR-TIRADS		EU-TIRADS		K-TIRADS	
	RoM (%)	Recommendation	RoM (%)	Recommendation	RoM (%)	Recommendation
1	< 2	No FNAC	–	–	–	–
2	< 2	No FNAC	≈0	No FNAC	< 3	No FNAC^b^
3	2.1–5	≥ 1.5 cm: follow-up ≥ 2.5 cm: FNAC	2–4	> 2 cm: FNAC	3–10	> 2.0 cm: FNAC
4	5.1–20	≥ 1 cm: follow-up ≥ 1.5 cm: FNAC	6–17	> 1.5 cm: FNAC	10–40	> 1–1.5 cm: FNAC^c^
5	> 20	≥ 0.5 cm: follow-up ≥ 1 cm: FNAC	26–87	< 1.0 cm: active surveillance^a^ > 1.0 cm FNC	> 60	> 1 cm: FNAC^d^

^a^The original EU-TIRADS guidelines report that “Patients with subcentimeter nodules with highly suspicious US features and no abnormal lymph nodes can have the choice of active surveillance or FNA”

^b^The original K-TIRADS guidelines report that FNAC “may be performed in nodules that demonstrate continuous and significant growth or for nodules prior to ablation therapy or surgery”

^c^The original K-TIRADS guidelines report that “Cutoff size for biopsy should be determined within the range of 1 and 1.5 cm, based on the ultrasound features, nodule location, clinical risk factors, and patient factors (age, co-morbidities, and preferences)”

^d^In the original, K-TIRADS guidelines reported that FNAC may be considered in smaller TNs in specific clinical contexts

According to the above issues, the present study aimed to explore prevalence and significance of the recommendation in favor of follow-up assessed according to ACR-TIRADS. With this aim, a retrospective histological series was collected. The results of EU- and K-TIRADS were also reported as comparator.

## Materials and methods

### Setting

Our institution is the public health institution of the region. It has the highest number of surgeries in that canton and all histological samples from surgeries performed in our region are evaluated at our pathology institute. Thus, the institutional database includes a large series of records of thyroid surgery and histology.

### Case selection

This study series was retrospectively collected according to a pre-defined 4-step protocol: (1) Search in the institutional database for records of patients undergoing thyroidectomy between 2019 January and June 2023; this phase was conducted by a data scientist blind of clinical data. (2) Inclusion of cases undergone pre-operative thyroid US with images available in PACS; this phase was performed by two radiologists blinded by surgical indication and histological findings. (3) Re-assessment according to ACR-, EU-, and K-TIRADS of TNs with major diameter of at least 5 mm; this phase was performed by the same two radiologists separately. (4) Matching of TIRADS and histological data; this phase was conducted by an expert clinician. Eventual discordant cases were solved in a mutual meeting among raters. This strategy was chosen in order to use histology as gold standard of the study.

### Statistical analysis

Continuous parameters were treated with non-parametric statistical analysis and are always expressed as median and interquartile range (IQR). Frequencies were analyzed by *χ*^2^ test. Diagnostic tests were calculated considering histological diagnosis as reference standard. Incidental micro-carcinoma was not considered for statistical analysis. The statistical significance was set at *p* < 0.05. Analyses and figures were performed with GraphPad Prim version 7 (GraphPad software, CA, USA).

## Results

### Case series and assessment of TNs according to ACR-TIRADS

Two-hundred-one TNs from 103 patients were initially found in the institutional database during the study period. One patient refused the study. After using the pre-defined inclusion criteria, 22 patients (median age 53 years, 17 females and five males) were selected for the study. Finally, the study series included 39 TNs assessed for follow-up according to ACR-TIRADS of which 10 (25.6%) cancers. Out of the latter, nine were papillary (PTC) and one medullary (MTC) carcinoma.

**Fig. 1:**
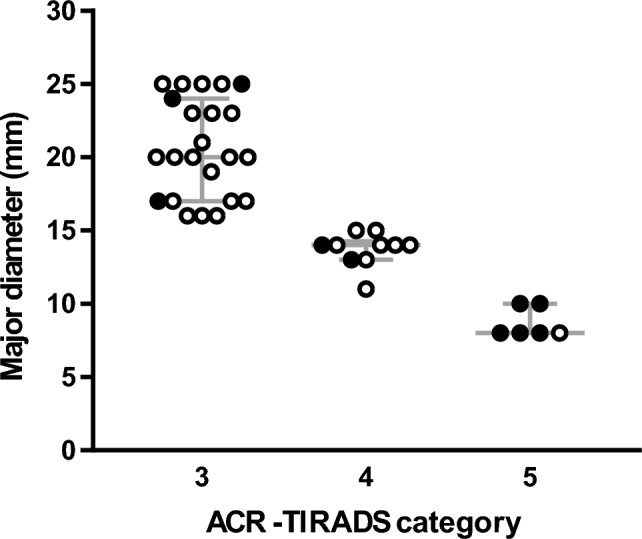
Distribution of the 39 TNs according to ACR-TIRADS category and dimension. Black point represents cancer and white point indicates benign lesions. Gray bars represent median and IQR

Among the 39 TNs, 23 were classified as category 3 of ACR-TIRADS, 10 as category 4, and 6 as category 5. As illustrated in Table 2, there were 13% and 20% of cancer rate among TNs assessed as category 3 and category 4, respectively. The cancer frequency among TNs assigned to category 5 was 83.3%. All PTCs were staged as low risk of relapse [2]. In addition to these figures, there were five small incidental papillary carcinomas, three in category 3, and one each in the other two categories (data not shown). Figure 1 illustrates the overall distribution of malignant and benign TNs according to ACR-TIRADS category and size.

**Table 2:** Characteristic and cancer outcome of the 39 TNs in which ACR-TIRADS indicates follow-up

ACR-TIRADS category	Cases, n	Size, median (IQR)	Cancer, n (%)	Cancer type	pTNM staging, n
3	23	20 (17–24)	3 (13)	PTC	pT2, 2 pT1bm, 1
4	10	14 (13–14)	2 (20)	PTC	pT1b, 2
5	6	8 (8–10)	5 (83.3)	PTC	pT1a, 2 pT1b, 2
				MTC	pT1a, 1

All cancers were staged according to the last edition of pTNM system. PTC, papillary thyroid carcinoma

*MTC* medullary thyroid carcinoma, *IQR* interquartile range

### Comparison between ACR-TIRADS and EU-TIRADS

When analyzing the re-assessment of the 39 TNs according to EU-TIRADS, one case was as category 2, 25 as category 3, eight as category 4, and five as category 5. From this point of view, no significant difference was observed between ACR- and EU-TIRADS (*p* = 0.70). Altogether, FNAC was recommended according to EU-TIRADS in 10 lesions. Among these, two were PTCs that were assessed as category 3 of ACR-TIRADS. In addition, EU-TIRADS indicated active surveillance in other three PTCs previously assessed as ACR-TIRADS 5. Overall, EU-TIRADS allowed to detect 2/10 (20%) cancers with eight unnecessary FNACs.

### Comparison between ACR-TIRADS and K-TIRADS

When the 39 TNs were re-assessed according to K-TIRADS, three cases were classified as category 2, 22 as category 3, nine as category 4, and five as category 5. No significant difference emerged in the distribution of TNs according to ACR- and K-TIRADS (*p* = 0.36). Altogether, FNAC was indicated according to K-TIRADS in 32 lesions. Out of the latter, seven were PTCs that were assessed as category 3 (*n* = 3), category 4 (*n* = 2), or category 5 (*n* = 2) according to ACR-TIRADS. Moreover, K-TIRADS assessed for selective FNAC other four cases (of which three cancers) that were assessed as category 5 according to ACR-TIRADS. Overall, K-TIRADS indicated correctly FNAC in 7/10 (70%) cancers with 25 unnecessary FNACs.

### Summary of results of ACR-TIRADS, and implications of using the other systems

**Fig. 2:**
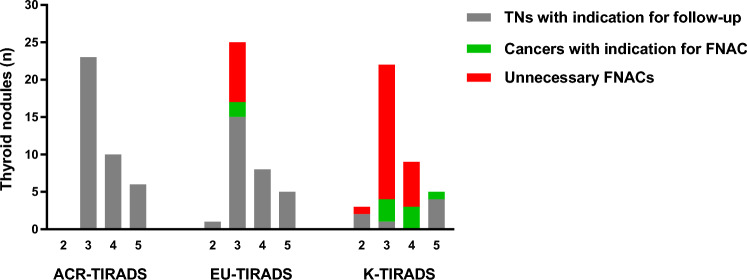
Assessment of the 39 TNs according to the three TIRADSs and their selection for FNAC. Where ACR-TIRADS does not indicate for FNAC, it actually assigns TNs to follow-up. Unnecessary FNAC is defined as biopsy performed in histologically-proven benign lesion

Overall, among TNs assigned to follow-up according to ACR-TIRADS, there was a cancer rate of 25.6%. FNAC indication according to EU-TIRADS allowed us to detect 20% of cancers with 80% frequency of unnecessary biopsies. K-TIRADS indicated correctly FNAC in 70% of cancers with the burden of 78.1% unnecessary FNACs. The rate of FNAC indication according to K-TIRADS was significantly higher than that observed according to EU-TIRADS (*p* < 0.0001). Figure 2 illustrates the absolute numbers of these results.

## Discussion

During the last years, TIRADSs have been largely and rapidly worldwide diffused. As a consequence, an increasing number of papers about TIRADS performance have been recorded [11] and several papers compared TIRADSs in terms of RoM assignment and FNAC indication. This matter is crucial in the current era since an international expert board is working to create the universal I-TIRADS [10]. The aim of the present study was to explore the subgroup of TNs in which ACR-TIRADS indicates for follow-up. As this kind of recommendation does not exist in the other TIRADSs, this issue should be interesting for clinicians, surgeons, and TIRADS users. The major results of the present study can be summarized as follows:

First, in a series of 103 patients who underwent thyroidectomy, there were 221 TNs that could be retrospectively assessed according to ACR-TIRADS, and 39 of them were assigned to follow-up. The frequency of TNs addressed to follow-up according to ACR-TIRADS was non-negligible and this should be taken into account for clinical practice.

Second, about one in four of TNs assigned to follow-up according to ACR-TIRADS was a cancer. Even if this finding looks like as alarming for TIRADS users, the cancers were low-risk PTC or micro-MTC. As small thyroid cancers are expected to have indolent behavior, and considering that ACR-TIRADS recommends to address those TNs to clinical observation, ACR-TIRADS users have to be fully aware of this data to better tailor the patient follow-up. This result is in line with that observed by Middleton et al. [12]. That study reviewed a series of 352 cancers and found that 72 (20.5%) cases were assessed for follow-up according to ACR-TIRADS; the largest part of cancers was assessed as category 5.

Third, when TNs are re-assessed according to EU- or K-TIRADS, some cancers could be detected by FNAC indication. Particularly, EU-TIRADS indicated FNAC in one-fourth of cases and detected one-fifth of cancers; instead, K-TIRADS recommended FNAC in three-fourth of TNs with a cancer detection of 70%. However, a significant difference emerged in terms of FNACs indication with higher rate according to K-TIRADS.

From the clinical point of view, a discussion of the present findings is then necessary. Thyroid cancers are frequent and often incidentally discovered. Generally, small-size cases have indolent behavior and most of them do not require radioiodine treatment [2]. Ultrasound and TIRADS have allowed us to significantly improve our selection of TNs for FNAC. Hence, TIRADS accuracy looks like similar to that of cytological evaluation [13]. However, first, TIRADS and ACR-TIRADS in particular were actually conceived to save on FNACs and its related costs and further implications (i.e., unnecessary surgery). Then, some thyroid carcinomas may skip the FNAC indication according to their small dimension. Searching for the optimal size threshold to indicate or not FNAC is a matter to debate, and several papers have been published on this topic [14–18]. Second, TIRADSs were built on the basis of PTC only and their accuracy has been proven against this cancer type [19]. Other cancers, such as follicular and medullary, may present at US with heterogeneous pattern, in any case different from that of PTC [20, 21]. As recorded in our study series, FNAC may not be indicated in MTC. These data raise the question of whether and how clinical concepts should be integrated in the risk stratification of TIRADSs. The future I-TIRADS should probably integrate clinical issues to further improve our accuracy. Whether I-TIRADS should include the indication for follow-up or FNAC in TNs with size just below that to recommend FNAC is challenging. Before achieving a consensus about this matter, the TN size thresholds above which actions (i.e., FNAC, follow-up, or other) have to be assessed, ideally after large prospective studies. In any case, the role of artificial intelligence should be clarified, and its impact in cancer detection rate needs to be better defined [22, 23].

Inevitably, the present findings achieve interest for surgeons. As the surgical approach to thyroid cancer has been changed during the last decade with increase of hemithyroidectomies, both endocrinologists and surgeons should take into account the presence of TNs in the contralateral thyroid lobe. On the one hand, when TNs are assessed for follow-up according to ACR-TIRADS, their risk of cancer is non-negligible with consequent non-negligible likelihood of re-operation in future. On the other hand, according to the present findings, cancers expected among TNs with indication for follow-up according to ACR-TIRADS are presumably at low risk of recurrence [2]. Then, planning hemithyroidectomy in selected cases (e.g., patients with cytologically indeterminate small TN in which ACR-TIRADS would indicate for follow-up) can be a reasonable first-line choice. This intrinsically means that thyroid surgeons become as much as possible aware of the TIRADSs. A multidisciplinary discussion remains necessary before planning the initial surgical treatment.

Limitations to the study and its strengths should also be discussed. This a retrospective series of patients that were managed according to their pre-operative clinical profile. Then, the retrospective re-assessment of TNs according to TIRADS should be seen as a potential bias. However, since we aimed to investigate TNs addressed to follow-up, this was the only strategy to evaluate their outcome. This setting cannot be retrieved in a series of FNACs. Moreover, the histological diagnosis avoids the methodological problem of dealing with indeterminate cytology that represents about 20% of FNACs. The retrospective interpretation of US images stored in RIS-PACS should be another bias. However, discordant assessment was solved with a mutual meeting to achieve a consensus.

## Conclusions

The present study demonstrated that TNs assessed for follow-up according to ACR-TIRADS are low-risk cancers in one-fourth of cases. EU- and, especially, K-TIRADS allow us to select for FNAC some of these cancers with the burden of non-negligible frequency of unnecessary FNACs. Clinicians and TIRADS users have to be aware of these findings. These figures can contribute to pave the way for developing the I-TIRADS. Before the introduction of I-TIRADS, surgeons should take into account the findings of the present study to better plan their interventions.

## Data Availability

The data sets used and/or analyzed during the current study are available from the corresponding author on reasonable request.
